# Pharmacological Fingerprinting of Cholesterol Biosynthesis Defects

**DOI:** 10.3390/biomedicines14102307

**Published:** 2026-10-11

**Authors:** Matija Fenrich, Ivan Šimunović, Marta Balog, Marija Heffer

**Affiliations:** 1 Department of Medical Biology and Getetics, Faculty of Medicine Osijek, J. J. Strossmayer University of Osijek, 31500 Osijek, Croatiaivan.simunovic35@gmail.com (I.Š.); mheffer@mefos.hr (M.H.); 2 Clinic for Psychiatry, Psychotherapy and Psychosomatic Medicine, AMEOS Klinikum Bremen, 28325 Bremen, Germany; 3 Department of Neurology, Institute of Neurophysiology, Klinikum Bremen-Ost, 28325 Bremen, Germany

**Keywords:** cholesterol biosynthesis, enzyme inhibitors, drug therapy, phenotyping, animal models

## Abstract

Cholesterol biosynthesis is an interconnected metabolic network that generates cholesterol as well as numerous sterol and non-sterol intermediates with essential structural, signaling and regulatory functions. While inborn defects of this pathway have traditionally been employed to study the functions of these metabolites, it has recently been demonstrated that numerous drugs used to treat common diseases inhibit individual enzymes within this network. Pharmacological inhibition of individual biosynthetic enzymes provides an opportunity to investigate pathway disruption in mature organisms without confounding effects associated with altered embryonic development. The effects of such inhibition—particularly in the context of polypharmacy—may have either therapeutically beneficial or adverse consequences and therefore require experimental validation in appropriate models. In this review, we examine commercially available and in vivo validated pharmacological inhibitors of cholesterol biosynthesis enzymes. We further introduce the concept of the pharmacological fingerprint, defined by the characteristic combination of precursor accumulation, downstream sterol depletion, pathway shunting and secondary metabolic adaptations produced by inhibition of a defined enzymatic step. By characterizing the fingerprints of each of these compounds, we demonstrate that pre-lanosterol inhibition primarily affects mevalonate-derived non-sterol isoprenoids and associated cellular functions, whereas post-lanosterol inhibition predominantly alters the distribution and accumulation of sterol intermediates. The available evidence supports pharmacological models as complementary tools to genetic models for dissecting the biological function of individual biosynthetic intermediates and for investigating drug-induced sterol disturbances beyond developmental disorders. However, differences in model fidelity, incomplete characterization of tissue-specific effects, off-target drug effects and inconsistent sterol measurements currently limit direct comparison between models. Future studies should therefore combine standardized sterol profiling with systematic tissue-specific phenotyping. Such an approach could help establish reference fingerprints and improve the experimental and translational utility of pharmacological models.

## 1. Introduction

Cholesterol is an essential lipid molecule with diverse structural and regulatory functions in mammalian physiology. Beyond its well-established role as a key component of cellular membranes, where it modulates membrane fluidity, permeability and the organization of signaling microdomains, cholesterol also serves as a precursor for steroid hormones, bile acids and signaling sterols, including oxysterols [1]. In the central nervous system (CNS), cholesterol is particularly abundant, accounting for nearly one quarter of the body’s total content, where it is indispensable for synaptogenesis, dendritic differentiation, myelination and neuronal plasticity [2,3,4]. Importantly, cholesterol metabolism differs substantially between peripheral tissues and the brain. While peripheral cholesterol homeostasis is maintained through a dynamic interplay of dietary uptake, hepatic synthesis and lipoprotein-mediated transport, brain cholesterol metabolism is largely autonomous due to the restrictive properties of the blood–brain barrier (BBB), necessitating de novo synthesis primarily by oligodendrocytes and astrocytes, and to a lesser extent by neurons [5,6,7,8].

Defects in cholesterol biosynthesis have been classically studied in the context of rare developmental disorders, where inherited mutations in biosynthetic enzymes lead to severe congenital malformations and neurodevelopmental abnormalities [9,10,11,12,13]. In adults, the role of excess cholesterol in the development of atherosclerosis has been thoroughly studied and clinically well-established. However, increasing evidence suggests that disturbances in cholesterol synthesis and metabolism may also contribute to conditions beyond cardiovascular diseases, including neurodegenerative and neuropsychiatric disorders, and (auto)immune pathologies, including tumors [1,2,6,14,15,16,17]. These observations warrant the expansion of the relevance of cholesterol homeostasis beyond the developmental window period.

Some commonly prescribed drugs have been shown to interfere with cholesterol biosynthesis [18,19,20,21,22]. This is a particular concern for compounds capable of crossing the BBB, where they may alter the tightly regulated sterol environment of the CNS. Despite the widespread clinical use of such medication, the potential consequences of chronic exposure, polypharmacy and individual genetic susceptibility (such as polymorphisms in cholesterol biosynthesis genes and heterozygous carrier state for pathogenic variants) remain insufficiently explored [23]. This knowledge gap may be especially relevant in vulnerable populations such as children, adolescents, pregnant women and possibly seniors.

Notably, some established cholesterol biosynthesis inhibitors, such as aripiprazole, are increasingly used and investigated in pediatric populations for various indications, such as tic disorders, Tourette syndrome and autism spectrum disorder [24,25,26]. This is particularly relevant, given that cholesterol synthesis in the developing brain remains elevated until late adolescence, reflecting the prolonged maturation of neuronal circuits and myelination [27,28]. The long-term consequences of pharmacological interference with the brain cholesterol metabolism during these critical developmental window periods, especially in the case of coexisting individual genetic susceptibility, remain unknown and warrant systematic investigation.

A recent comprehensive review by Peeples et al. [22] systematically cataloged chemical compounds capable of interfering with certain post-lanosterol cholesterol biosynthesis enzymes. However, the utility of pharmacological inhibition as a strategy for generating experimentally defined, in vivo models of cholesterol biosynthesis defects across the entire pathway has not yet been systematically examined. The present paper aims to bridge this gap by reviewing the current landscape of commercially available and in vivo validated pharmacological compounds capable of inducing cholesterol biosynthesis disruption in animal models.

We use the term pharmacological fingerprint to denote the characteristic pattern of sterol depletion, precursor accumulation, pathway shunting and secondary metabolic adaptations generated by inhibition of defined enzymatic steps in the cholesterol biosynthesis network. Importantly, the term describes a biochemical signature rather than an assumption of phenotypic equivalence to a corresponding genetic defect. The extent to which a certain pharmacological intervention recapitulates a corresponding sterolopathy is referred to in this paper as model fidelity.

Furthermore, by integrating the site of enzymatic inhibition with the resulting accumulation or depletion of biologically active pathway intermediates and the observed systemic and tissue-specific phenotypes, we propose that the pharmacological inhibitors discussed in this paper represent a practical and, as of today, still insufficiently employed tools to study the pleiotropic biological functions of different cholesterol biosynthesis intermediates, with a caveat that careful distinction between effects caused by the enzymatic block, secondary metabolic remodeling and compound-specific off-target pharmacology should be made.

These compounds could further serve to establish reference models for development and validation of future inhibitors and/or biosynthesis modulators, and to aid the molecular phenotyping of (side) effects of drugs, whose impact on cholesterol biosynthesis enzymes is still understudied. We further aim to provide a conceptual framework for future studies and identify critical gaps in the current knowledge. We hope that this could improve the risk stratification and therapeutic safety in vulnerable populations taking drugs interfering with cholesterol biosynthesis.

## 2. Materials and Methods

The objective of this review was not to provide an exhaustive systematic synthesis of all available studies but rather to identify in vivo validated pharmacological models across different steps of the cholesterol biosynthesis pathway and discuss their biochemical and phenotypic fingerprints. Therefore, the literature selection was guided by relevance to the conceptual framework of the review. We searched the bibliographic databases PubMed and MEDLINE for animal studies involving pharmacological inhibition of enzymes involved in cholesterol biosynthesis. Electronic databases were searched with no temporal constraints applied to the literature. Search strings were constructed using the enzyme name or its standard abbreviation, alongside keywords for inhibition and in vivo context. Four independent researchers (M.F., I.Š., M.B. and M.H.) screened the abstracts for eligibility. Studies involving transgenic cholesterol biosynthesis effects, as well as in silico and in vitro studies were manually excluded. In silico studies, although valuable for integrating big data [29], were excluded because the present review focuses on experimentally validated in vivo biochemical models. In vitro studies were mentioned in the context of development of the bioactive compounds, yet excluded from further discussion as the paper focuses on validated in vivo animal models, and also because in vitro studies lack the biological complexity inherent to cholesterol biosynthesis, altered pharmacokinetics, and limitations in translational relevance [30]. In that light, we emphasize that some compounds that showed inhibitory properties in vitro, failed to do so in vivo, as recently demonstrated in studies screening for inhibitors of 7-dehydrocholesterol reductase [18]. Furthermore, because this review focuses on the consequences of sterol synthesis inhibition after organogenesis, studies using three-dimensional organoids derived from human stem cells were also excluded. Genetic interventions targeting sterol metabolism at the zygote stage were mentioned, but not discussed in depth due to the well-known impact of sterol deficiency on developmental processes [31] and the establishment of an altered baseline physiology. “Just-in-place” genetic models were excluded because their effects are directed at a targeted organ, unlike the systemic action of pharmacotherapy. Any disagreement or ambiguity regarding the inclusion was resolved by advancing the study to a full-text review.

## 3. Pharmacological Fingerprints of Pre-Lanosterol Cholesterol Biosynthesis Defects

In the developmental paradigm, most animal models center around the so-called post-lanosterol defects of cholesterol biosynthesis, because the total biosynthesis blockage in the proximal, pre-lanosterol segment of the pathway during early development is commonly not compatible with life [32,33]. On the other hand, the defects in the pre-lanosterol enzymes in adult organisms lead to less drastic consequences.

**Figure 1:**
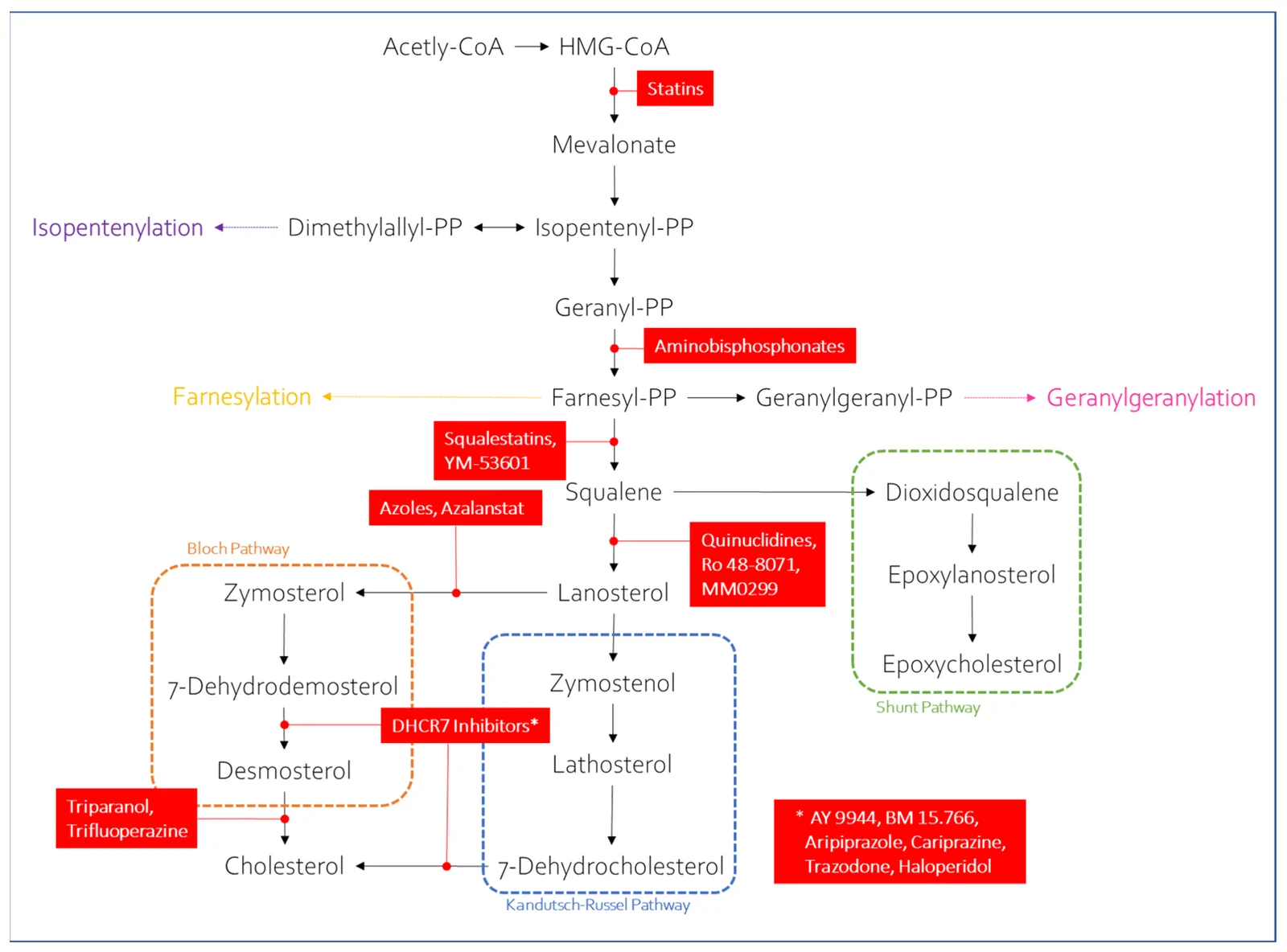
Simplified scheme of cholesterol biosynthetic pathways with regard to the most established biochemical inhibitors used in animal models (depicted in red boxes). The main functional branches of the cholesterol biosynthesis network include isopentenylation (post-transcriptional modification of tRNAs, ensuring stability during ribosome-binding), farnesylation (post-translational modification essential for membrane anchoring, protein–protein interactions and activation of small GTPases involved in cell cycle regulation), geranylgeranylation (post-translational modification involved in targeting and trafficking of proteins and vesicles to specific subcellular compartment), shunt pathway (sometimes called Popják pathway, prevents the overaccumulation of isoprenoids by diverting the mevalonate metabolites into non-sterol pathways, i.e., fatty-acid metabolism and energy production), and Bloch and Kandutsch–Russel pathways (final reactions, leading to biosynthesis of cholesterol and yielding oxysterols, potent signaling molecules involved in cholesterol homeostasis, immunomodulation, morphogenesis, cell survival and apoptosis). The earlier the inhibition in enzymatic steps occurs, the more cellular functions are being jeopardized.

The pre-lanosterol segment, extending from acetyl-CoA through the mevalonate pathway to lanosterol, constitutes a central metabolic hub that generates not only sterol precursors, but also essential non-sterol isoprenoids. These include substrates required for protein prenylation, ubiquinone biosynthesis, dolichol-mediated N-glycosylation, heme A production and tRNA isopentenylation [34] (Figure 1). Consequently, deficiency or inhibition of enzymes in this proximal pathway disrupts multiple vital cellular processes. Biallelic knockout of genes encoding enzymes involved in the early mevalonate-pathway results in early embryonic death in mice [32,35]. Human embryos harboring biallelic null variants for these genes are also non-viable, with a notable exception of mevalonate kinase deficiency, which is usually associated with hypomorphic alleles, thus retaining some partial enzymatic activity [36,37].

By contrast, post-lanosterol defects largely preserve non-sterol isoprenoid synthesis, allowing embryonic survival despite impaired cholesterol production and accumulation of toxic sterol intermediates. This residual metabolic integrity results in typical, partially overlapping malformation syndromes, featuring a more or less pronounced intra-uterine growth restriction with microcephaly, craniofacial dysmorphisms, limb anomalies and moderate to severe intellectual disability, often exhibiting clinical similarity to autism spectrum disorder [38,39,40,41].

In adult organisms, however, inhibition of pre-lanosterol biosynthesis is not immediately lethal, but can lead to consequences that extend far beyond simple cholesterol depletion [22] because, as previously mentioned, this proximal mevalonate pathway also supplies essential isoprenoids. The phenotype therefore most likely depends on the degree, duration and tissue specificity of the inhibition.

As shown in the following paragraphs, each enzymatic block produces a characteristic pattern of substrate accumulation and downstream sterol depletion that can serve as a biochemical fingerprint of the corresponding biosynthetic defect. These fingerprints are not necessarily restricted to the immediate substrate of the inhibited enzyme: accumulated sterols may enter shunt pathways [42], undergo oxidation [43], alter membrane properties [44] and intracellular signaling [45], or exert feedback effects on upstream enzymes [46]. Consequently, the phenotype generated by a pharmacological inhibitor represents the combined result of the primary enzymatic block, compensatory metabolic responses and the biological activity of accumulating intermediates.

The most well-established animal models of pre-lanosterol biosynthetic defects employ the inhibition of 3-hydroxy-3-methylglutaryl-co-enzyme-A-reductase (HMGCR), enzymes downstream to mevalonate kinase (MVK) and mevalonate decarboxylase (MVD), squalene synthase (FDFT1), squalene epoxidase (SQLE) and lanosterol synthase (LSS). As discussed hereafter, the common denominator of these models are dose-dependent, complex multisystem effects driven primarily by the loss of non-sterol isoprenoid homeostasis, rather than cholesterol deficiency alone. Moreover, these effects are yet to be tissue-specifically categorized.

### 3.1. HMGCR Inhibition: The Mevalonate-Depletion Fingerprint

Statins are frequently prescribed cholesterol-lowering drugs that exert their action by inhibiting HMGCR, an important and rate-limiting enzyme in the pre-lanosterol segment of cholesterol biosynthesis. By also interfering with isoprenoid biosynthetic pathways, they were found to exert initially unanticipated pleiotropic effects on different tissues, conferring benefit to cardiovascular risk reduction [47]. At the same time, they are being criticized for their unspecific and broad interference with these vital cellular processes [48]. The partial pharmacologic inhibition with statins is reported to be acceptably tolerated, most likely because residual, non-inhibited enzymatic activity usually remains sufficient to sustain essential isoprenoid production.

However, stronger and possibly prolonged inhibition reduces availability of farnesyl-pyrophoshate (FPP) and geranylgeranyl-pyrophosphate (GGPP), impairing protein prenylation of small GTPases, such as Ras, Rho and Rab, possibly leading to altered cytoskeletal dynamics, vesicular trafficking and impairments of transmembrane signal transduction [49,50].

Seshadri et al. found that chronic treatment with atorvastatin and simvastatin impairs insulin signaling cascade in rats fed with a high-sucrose diet, resulting in worsening of glucose intolerance and hyperglycemia [51]. Another group demonstrated a dose-dependent insulin-signaling impairment in a model of chronic fluvastatin treatment of obese mice [52], further corroborating the potential deleterious effects of prolonged HMGCR inhibition on receptor signaling.

Reduced synthesis of ubiquinone as a consequence of HMGCR inhibition may compromise mitochondrial electron transport and thus ATP generation, contributing to fatigue, myopathy, and in severe cases, rhabdomyolysis [53]. In 2001, cerivastatin was withdrawn from the market due to deaths related to rhabdomyolysis [54].

Depletion of dolichols can impair N-linked glycosylation, disrupting essential posttranslational modifications of various membrane proteins. In immune cells, defective prenylation has been shown to activate inflammasome and promote excess interleukin-1β release, a pathological mechanism also implicated in mevalonate kinase deficiency [55,56].

In proliferative tissues, profound inhibition of HMGCR may suppress cell-cycle progression or induce apoptosis. For this and all the above-mentioned reasons, there are attempts to repurpose the proximal mevalonate-pathway blockade into oncological chemotherapy [57,58,59]. For the extensive review of the current research progress on the cholesterol biosynthesis modulation in the context of the tumor therapy, we kindly recommend consulting the paper by Zewen et al. [60].

Unlike in embryogenesis, adult tissues seem to be able to temporarily buffer cholesterol deficits through existing reserves, dietary uptake and lipoprotein recycling [61,62]. Nevertheless, sustained or near complete pre-lanosterol inhibition in animal models is typically cytotoxic, particularly in metabolically active tissues such as skeletal muscle, liver and the brain [16,63].

The current evidence regarding the effects of statins on cognitive function in animal models is far from conclusive. A discrepancy in relation to human studies, which overwhelmingly report beneficial effects of statin therapy on brain functions is also noteworthy. While inhibition of protein prenylation in the CNS has been hypothesized to vest neuroprotective effects [64], and a meta-analysis of acute stroke patients revealed that acute, high-dose therapy with statins improve the functional outcome [65], several independent studies on rodents showed that chronic treatment with lipophilic statins (that readily cross the BBB) impairs hippocampal synaptic plasticity, working and recognition memory, whereas drug discontinuation restores the observed deficits [66,67,68]. Therefore, when accounting for the effects on the CNS, we propose that a distinction between hydrophilic and lipophilic statins should be considered.

Hydrophilic statins (rosuvastatin and pravastatin) do not cross the BBB and require active transport across the cell membranes, making them more liver-selective compared to their lipophilic counterparts (atorvastatin, simvastatin, lovastatin, fluvastatin and pitavastatin) [69]. The development of tissue-specific, nanoparticle-mediated drug delivery systems might therefore help to maximize therapeutic efficacy at disease sites while minimizing systemic toxicity [70].

Ultimately, one must bear in mind that an inhibition of a certain enzyme in a biosynthetic pathway does not only imply a depletion of its product(s), but also an accumulation of the precursors. In the case of the HMGCR inhibition, this would imply a build-up of 3-hydroxy-3-methylglutaryl-coenzyme A (HMG-CoA), as it was recently demonstrated both in vivo as well as in vitro by Trub et al. [71]. Historically, observing this specific molecular accumulation in live tissue was technically difficult due to the highly unstable (i.e., highly reactive) nature of acyl-CoA molecules. However, modern mass spectrometry and molecular tracking have thoroughly documented this effect. Trub et al. showed that the high reactivity of HMG-CoA is not trivial, since accumulated HMG-CoA leads to a non-enzymatic modification, termed HMGylation. So far, fatty-acid synthase, an enzyme involved in lipid biosynthesis, has been identified as a target of HMGylation, leading to its inhibition and altered cellular signaling around the endoplasmic reticulum and Golgi apparatus [71].

In conclusion, the currently available data from in vivo studies suggest that a HMGCR inhibition leaves a biochemical fingerprint consisting not only of cholesterol depletion, but also at least partial depletion of all downstream sterols and intermediates involved in vital cellular processes, including isopentenylation, farnesylation and geranyl-geranylation, as well as a fingerprint of HMG-CoA accumulation, and still poorly understood and researched protein HMGylation (Table 1). This fingerprint overlaps significantly with the biochemical pattern observed in both monoallelic knockouts for HMGCR and the autoimmune condition termed anti-HMGCR myopathy [72,73].

### 3.2. MVK/MVD Inhibition: The Mevalonate-Congestion Fingerprint

Unlike the HMGCR inhibition, which leads to partial depletion of all the downstream intermediates in the biosynthetic pathway, the inhibition of post-mevalonate and pre-lanosterol steps additionally causes toxic accumulation of mevalonate and phosphomevalonate. The phenotype is therefore characterized not only by perturbations in FPP, GGPP, ubiquinone and dolichol homeostasis, but also by recurrent proinflammatory crises, mediated by amplification of innate immune responses by accumulating mevalonate intermediates [36,37,56].

In humans, complete MVK deficiency results in non-viable embryos, whereas individuals retaining partial enzymatic activity exhibit a malformation syndrome with concomitant severe immune dysregulation, termed hyperimmunoglobulinemia D with periodic fever [36,37].

Bifunctional inhibitors of MVK and MVD have been synthesized [74,75,76]. At present there are, however, no established animal models of pharmacologic MVK/MVD inhibition. All available in vivo studies employed genetically modified animals [35,77,78].

The up to date best-established biochemically induced animal model, developed by Marcuzzi et al., relies on inhibition of farnesylpyrophosphate-synthase instead, an enzyme just downstream of MVK/MVD, by treating wild-type mice with aminobisphosphonates such as pamidronate and alendronate, which is reported to approximately reproduce the phenotype of MVK deficiency [79,80,81]. In humans, these drugs are used in diseases that negatively affect bone mineralization, such as osteoporosis, Paget disease or various tumor-induced bone pathologies, where they inhibit bone-resorbing osteoclasts by disrupting their protein prenylation [82]. Notable difference in this animal model in relation to the direct MVK/MVD deficiency might be that the inhibition via aminobisphosphonates should theoretically spare isopentenylation (Figure 1). Moreover, bisphosphonates do not cross the BBB, as demonstrated in vivo in rodents, canines and rabbits [83,84], implying that this model is likely not able to reproduce possible CNS effects of mevalonate congestion.

Thus, the so-far reported in vivo biochemical fingerprint exerted by aminobisphosphonates can be summarized as depletion of downstream sterols, non-sterol isoprenoids and a build-up of mevalonate, precipitating detrimental proinflammatory crises (Table 1).

### 3.3. FDFT1 Inhibition: The Isoprenoid-Accumulation Fingerprint

Inhibition of cholesterol biosynthetic pathways downstream to squalene preserves the geranylgeranylation, farnesylation and isopentenylation, while depleting the intermediates of the Bloch, Kandutsch–Russel and the shunt pathways (Figure 1). This is the common feature of inhibitors of squalene synthase (FDFT1), squalene epoxidase (SQLE) and lanosterol-synthase (LSS).

The most-researched FDFT1 inhibitors as of today are squalestatins, also known as zaragozic acids. Unlike HMGCR and immediate post-MVK/MVD inhibitors, squalestatins are considered sterol-selective pre-lanosterol inhibitors [85]; in other words, they are not interfering with non-sterol isoprenoid biosynthesis, at least not directly.

In rats and marmosets, these compounds showed very potent effects on serum cholesterol reduction without the myotoxic or inflammatory phenotype seen with proximal inhibition [86]. However, the early studies conducted on rodent liver and brain homogenates and ovary cell culture demonstrated a pronounced accumulation of pre-squalene intermediates, in particular of FPP, dolichol and ubiquinone [87]. The further research on squalestatins was brought to a halt in 2011 due to hepatotoxicity concerns that emerged during the advanced stage of a phase III trial with a candidate drug, lapaquistat. The drug also showed less robust LDL reduction compared to statins [88].

Nonetheless, a recent study examined a novel squalestatin, 4-methyl-2-(10H-phenothiazin-3-yl)morpholin-2-ol hydrobromide in *Apoe* ^−/−^ mice, a transgenic model of atherosclerosis, with promising effects. The long-term administration of this compound significantly reduced atheromatic lesions and improved the glycemia in mice [89]. As with many other cholesterol biosynthesis inhibitors, some squalestatins are currently being considered for repurposing into chemotherapeutic agents [90,91].

In the search of an equally potent, yet pharmacokinetically more favorable FDFT1 inhibitor, the compound called YM-53601 was synthesized. This inhibitor was shown to be mechanistically similar to squalestatins, depleting post-squalene intermediates including cholesterol, while preserving upstream mevalonate flux, protein prenylation, dolichol synthesis and even increasing the production of ubiquinone [92]. In rats, YM-53601 showed a robust inhibition of de novo cholesterol biosynthesis [93], and it substantially reduced the non-HDL cholesterol in guinea pigs and rhesus monkey [94].

Surprisingly, YM-53601 strongly reduced triglycerides in hamster, even outperforming fenofibrate in some models [92,94], which was unexpected for a cholesterol-pathway inhibitor. This finding once again clearly demonstrated that inhibiting a single step in the cholesterol biosynthesis rarely leads to an isolated reduction in cholesterol, because all of the intermediates down the pathway are also biologically active signal molecules on their own. In the case of YM-53601, the secondary accumulation of FPP was identified to alter hepatic lipid flux and SREBP-linked regulation, leading to triglyceride depletion [92].

The further pharmacological development of cholesterol-lowering drugs based on YM-53601 was abandoned, as animal studies revealed that FDFT1 inhibition leads to the accumulation of toxic farnesyl-derived dicarboxylic acids as well [95].

Yet, the biomedical potential of FDFT1 inhibitors still lives on. Recent studies on rats and mice, that examined differentially expressed genes after ischemia, found a significant upregulation of *Fdft1*, the gene encoding squalene synthase, suggesting that FDFT1 inhibition might emerge as a promising target modulating the outcome of acute hypoxic injury [96,97]. In a mouse model of a middle cerebral artery occlusion–reperfusion, the gene was shown to be upregulated both in the liver and in the brain of animals following ischemia [97].

In summary, the current in vivo studies revealed that the biochemical fingerprint of FDFT1-inhibition can be summarized as partial depletion of downstream sterols, concomitant accumulation of FPP, and thus also of dolichol and ubiquinone, leading to altered hepatic gene transcription and secondarily to reduction in triglycerides synthesis (Table 1). This partially overlaps with the biochemical profile observed in cell cultures with genetic FDFT1 downregulation [98,99]; yet, the corresponding in vivo data obtained from haploinsufficient organisms are still lacking.

### 3.4. SQLE Inhibition: The Squalene-Accumulation Fingerprint

SQLE is a key, rate-limiting enzyme, catalyzing the conversion of squalene in 2,3-epoxysqualene, which are then further converted towards lanosterol [100]. By applying the analogy from the previously discussed inhibitors, one could expect that the SQLE inhibition leaves a cellular mark of depletion of downstream products and build-up of the immediate intermediates, i.e., squalene (Figure 1).

For years, the most-researched SQLE inhibitor was terbinafine, an antifungal drug that strongly binds to fungal SQLE, leading to ergosterol depletion at therapeutic doses. It should be noted that the biosynthetic pathway for sterols in fungi culminates in ergosterol, rather than in cholesterol as it does in animals. Terbinafine, however, in contrast to the fungal homologues, binds to mammalian SQLE much less avidly, and shows a very modest inhibition in vivo [101,102,103]. In the 1980s the quinuclidine derivatives of terbinafine were synthesized, initially focusing on their antifungal properties. However, it soon became clear that quinuclidines are also potent, competitive mammalian SQLE inhibitors [104], redirecting the research towards their potential cholesterol-lowering properties. The most-researched quinuclidine derivatives were named NB-598 and FR194738. Single oral dosing of NB-598 in rats inhibited hepatic cholesterol synthesis and repeated dosing in dogs lowered the total and LDL-cholesterol [105]. FR194738 lowered plasma cholesterol and triglyceride levels in dogs and hamsters, but, surprisingly, not in rats [106,107].

As predicted, it also led to a marked rise in squalene serum concentration [105]. The further studies on candidate drugs NB-598 and FR194738 were also stopped due to accumulation of squalene in the skin [108]. Nonetheless, squalene is safely used as an adjuvant in human vaccines and as an emollient in various cosmetic products for years, so far without indication of potential toxicity [109,110].

However, the effects of a systemic accumulation of squalene, as expected following pharmacological SQLE inhibition, are still inconclusive. The so-far available in vivo animal studies show conflicting results: pigs fed a squalene-supplemented diet exhibited alterations in gene networks that correlate to non-alcoholic fatty-liver disease [111], and, in a yeast model, intracellular accumulation of squalene was demonstrated to be lipotoxic if a cell cannot efficiently store excess lipids [112]. On the other hand, studies in mice and rabbit revealed rather beneficial cardiovascular and oxidative effects [113].

Setting the conflicting results aside, it is still important to point out that squalene is not just a simple structural precursor to cholesterol and steroids, but also a bioactive signaling and regulatory molecule in animals. It has been implicated in modulation of immune cell activation, altering transcription of genes involved in lipid homeostasis and buffering of oxidative stress [114,115,116].

Thus, the biochemical fingerprint of SQLE inhibition reflects a relatively preserved non-sterol isoprenoid synthesis on the one side, and a still poorly understood and characterized systemic accumulation of squalene on the other side (Table 1). The corresponding genetic models show a similar pattern of preserved isoprenoids, yet with an important difference: homozygous loss of *Sqle* led to extensive accumulation of squalene, whereas no significant systemic or pathogenic squalene accumulation was observed in heterozygous knockouts [117,118].

### 3.5. LSS Inhibition: The Epoxysterol Fingerprint

Inhibition of LSS, the enzyme responsible for conversion of 2,3-oxidosqualene to lanosterol, does not only lead to lanosterol depletion, but also to accumulation of pre-lanosterol substrates, which are then in turn being increasingly diverted to the shunt pathway (Figure 1). This exact biochemical fingerprint is attested with Ro 48-8071, a potent and in vivo validated inhibitor of LSS [42,119,120].

Diverting the pre-lanosterol intermediates into the shunt pathway was shown to increase the production of epoxysterols [42], active signaling molecules that in turn negatively influence the transcription of gene coding for HMGCR and accelerate its ubiquination and endoplasmic reticulum-associated degradation [121,122]. By means of this regulatory feedback loop, Ro 48-8071 indirectly exerts effects similar to statins.

Yet unlike statins, Ro 48-8071 did not negatively affect the synthesis of ubiquinone in hamster and was found to be at least as efficacious as simvastatin in lowering cholesterol in vivo in hamsters, squirrel monkeys and Göttingen minipigs [42]. Curiously, a study examining the sustained LSS inhibition by Ro 48-8071 in mice found that after an initial period of systemic cholesterol synthesis inhibition, the hepatic cholesterol production rebounded to higher than baseline rates within a week, whereas the cholesterol synthesis in the intestines and stomach remained suppressed [119]. At present, no study reporting on BBB penetrance of Ro 48-8071 could be retrieved.

Contrary to that, another highly selective and in vivo validated small-molecule LSS inhibitor, known as MM0299, was recently shown to be orally bioavailable and readily brain-penetrant, at least in mice [123]. This substance was studied as a potential novel therapeutic agent for glioblastoma, a highly malignant brain tumor. The LSS was chosen as a target since its inhibition does not only lead to cholesterol depletion, but it also secondarily increases the concentration of epoxysterols in the same way as Ro 48-8071 does, thus additionally boosting the tumor cholesterol depletion via the aforementioned negative feedback loop [123].

This precisely sums up the biochemical fingerprint of LSS inhibition: downstream sterol depletion with concomitant pre-lanosterol intermediates congestion (such as 2,3-oxidosqualene), which are consequently being redirected to the shunt pathway, leading to systemic accumulation of epoxysterols, bioactive molecules that reduce the HMGCR activity (Table 1). The very same fingerprint was reported in a cell culture with monoallelic knockout for the gene coding for LSS [124]. However, the developmental studies on these compounds are lacking. Therefore, it is not known if this pharmacological intervention might reproduce the phenotype usually seen with a biallelic loss of *Lss*, resulting in complex, yet variable clinical presentations with cognitive disabilities, hypotrychosis and cataracts [125].

## 4. Pharmacological Fingerprints of Post-Lanosterol Cholesterol Biosynthesis Defects

In contrast to proximal mevalonate-pathway inhibition, post-lanosterol inhibition is expected to initially preserve the synthesis of non-sterol isoprenoids, thereby shifting the principal metabolic disturbance toward sterol redistribution and accumulation (Figure 1).

From this perspective, pharmacological models of post-lanosterol defects can be distinguished according to their predominant sterol signature: lanosterol-14α-demethylase (CYP51A1) inhibition produces accumulation of lanosterol and 14α-methyl-sterols, 7-dehydrocholesterol reductase (DHCR7) inhibition shifts the sterol pool toward 7-dehydrocholesterol (7-DHC) and related Δ7-sterols, and 24-dehydrocholesterol reductase (DHCR24) inhibition increases desmosterols and other Δ24-unsaturated sterols.

While chemical inhibitors of post-lanosterol enzymes have been extensively reviewed elsewhere [22], we focus here solely on the compounds which are validated in vivo throughout a substantial body of experimental studies, with the focus on the aforementioned biochemical fingerprints and biological consequences of intermediate depletion and/or accumulation.

### 4.1. CYP51A1 Inhibition: The Lanosterol/14α-Methyl-Sterol-Accumulation Fingerprint

CYP51A1 inhibitors seem to be suitable for modeling the post-lanosterol defects in cholesterol biosynthesis (Figure 1). Similar to SQLE-inhibitors, CYP51A1 inhibition is expected to preserve protein prenylation, dolichol and ubiquinone biosynthesis and avoid mevalonate congestion. However, reduced CYP51A1 activity correlates in vivo with accumulation of lanosterol and other 14α-methyl-sterols [126,127], biologically active intermediates capable of perturbing membrane dynamics, signaling, hepatic lipid handling and meiosis initiation [128,129]. Moreover, 14α-methyl-sterols, similarly to previously discussed epoxysterols, are known to negatively influence the transcription of HMGCR [46,130]. Therefore, it is at least theoretically feasible that sustained CYP51A1 inhibition could indirectly reduce the cellular pool of non-sterol isoprenoids by means of this negative feedback loop. Curiously, we could not retrieve a single study that explicitly examined the long-term effects of HMGCR downregulation via CYP51A1 inhibition.

Abnormal expression of CYP51A1 is reported to be associated with carcinogenesis and various human metabolic, (auto)immune and neurodegenerative diseases [131,132]. Therefore, the inhibition or modulation of CYP51A1 activity was theorized to be a beneficial therapeutic target in various human health conditions [131,133] worth further testing on animal models.

The first group of in vivo validated and commercially readily available CYP51A1 inhibitors are azoles, antifungal drugs that bind the heme iron of CYP51A1 via nitrogen coordination and thus inhibit its enzymatic function [134]. Fluconazole is considered a gold standard in treatment and prophylaxis of candidiasis, with voriconazole and itraconazole as second-line options [135]. Although optimized for fungal CYP51, they show cross-reactivity with mammalian homologues [136]. An in vivo study of ketoconazole on rats found that six-times higher doses are required to influence the liver cholesterol synthesis compared to doses required to inhibit the fungal synthesis of ergosterol [137].

Azoles, as many other antifungal agents, are notorious for their hepatotoxicity [138]. It has been proposed that hepatotoxic effects after azole exposure are driven by altered expression of genes involved in cholesterol synthesis [138]. Recently, Takahashi et al. showed that certain CYP51A1 polymorphisms might confer protection against voriconazole-induced hepatotoxicity [139]. In cells, animal models and humans, the inhibition of CYP51A1 causes accumulation of lanosterol and other 14α-methyl-sterols [140,141], which are, as previously mentioned, biologically active compounds. Furthermore, analog to the LSS inhibition, the inhibition of CYP51A1 with azoles might also cause a similar increase in the flux of pre-lanosterol intermediates into the shunt pathway, leading to increased synthesis of epoxysterols. This effect, however, has at present only been reported in vitro [142].

While some azoles, such as fluconazole and voriconazole, permeate the BBB very well [143,144], others do so poorly or not at all [145,146,147]. In a study conducted on mice treated intraperitoneally with voriconazole, significantly reduced levels of cholesterol, squalene and lathosterol in the brain were found, as well as a significant decrease in transcription of HMGCR [148]. These results are pivotal, since they not only confirm the capability of these drugs to perturb the cholesterol synthesis in the CNS, but, also, as indirectly demonstrated by reduction in HMGCR, they corroborate the possibility that azoles indeed might engage the negative feedback loop via epoxysterol shunting.

In addition, miconazole and clobetasol were recently also found to cross the BBB and, curiously, were reported to enhance remyelination in mouse models of multiple sclerosis. It is hypothesized that this effect arises through the epoxysterol-mediated stimulation of oligodendrocyte differentiation [149,150]. Considering the current body of evidence, spanning across several different brain-penetrating azoles, the available data suggest that azoles also promote the spillage of pre-lanosterol intermediates into the shunt pathway.

Besides azoles, another established compound that shows robust cholesterol-lowering effects in vivo by inhibiting CYP51A1 is a synthetic imidazole, azalanstat, initially also known as RS-21607 [130]. In hamsters, a single 30 mg/kg oral dose successfully inhibited hepatic cholesterol biosynthesis by 50%. The same study also demonstrated the systemic accumulation of lanosterol and its immediate intermediates [151]. Furthermore, Morisawa et al. found that azalanstat in high doses efficiently reduced the production of bilirubin in mice by inhibiting heme oxygenase [152]. The further development of azalanstat was halted early in the preclinical phase in 1999, likely because of the concomitant “statin boom”, and the aforementioned unexpected effect on the heme oxygenase, restricting the live animal testing to rodents.

In brief, the current in vivo studies on CYP51A1 inhibition reveal a biochemical fingerprint consisting of partial downstream sterol depletion accompanied by accumulation of lanosterol and 14α-methyl-sterols, as well as increased shunting towards epoxysterols. The resulting build-up of epoxysterols might, in case of a sustained inhibition, progressively remodel the upstream pathway through sterol-mediated feedback, thus potentially converting an initially selective post-lanosterol perturbation into a broader metabolic phenotype.

This might be in accordance with findings from genetically modified animals: while biallelic knockout for *Cyp51a1* leads to embryonal lethality [153], monoallelic knockout organisms typically initially do not present overt clinical phenotypes; however, in the long term, they harbor sex-specific metabolic pathologies—males exhibiting a marked predisposition to hepatomegaly and hypercholesterolemia, and females to elevated plasma corticosterone [154].

### 4.2. DHCR7 Inhibition: The 7-DHC-Accumulation Fingerprint

DHCR7 is the enzyme responsible for the final step of cholesterol biosynthesis, catalyzing the conversion of 7-dehydrocholesterol (7-DHC) to cholesterol, as well as the reduction of 7-dehydrodesmosterol (7-DHD) to desmosterol. By acting at this late step of the biosynthetic pathway, DHCR7 inhibition preserves the metabolic flux toward essential non-sterol isoprenoids, ensuring that the biosynthesis of dolichol and ubiquinone, as well as protein prenylation, remains intact. As implied by Figure 1, this inhibition would be expected to cause accumulation of pre-7-DHC intermediates. The characteristic biochemical signature of DHCR7 inhibition is a metabolic shift from cholesterol toward 7-DHC, the most oxidizable sterol, and related sterol intermediates [43]. These highly reactive species are collectively termed 7-DHC-derived oxysterols, and they exhibit a plethora of signaling and direct biological effects, interfering with immunomodulation, lipid homeostasis, embryonal development, cell cycle regulation and cell survival [155,156].

Apart from the effects exerted by 7-DHC-derived oxysterols, it is important to understand that 7-DHC is also an active signaling modulator on its own: it was shown to suppress canonical TGF-β signaling, inhibit the Wnt/β-catenin pathway, to alter neuronal differentiation, to act locally as an endogenous antioxidant, to suppress phospholipid peroxidation and to inhibit ferroptosis [157,158,159]. Moreover, 7-DHC is a biosynthetic precursor of vitamin D synthesis in the skin [160].

In some cases, the alterations caused by DHCR7 inhibition closely resemble the biochemical profile of Smith–Lemli–Opitz syndrome (SLOS), sometimes even leading to the misdiagnosis of this rare autosomal recessive disorder in fetuses of treated pregnant women [161,162].

The current landscape of commercially available DHCR7 inhibitors can be broadly classified into two groups: classical experimental compounds, such as AY-9944 and BM 15.766, and clinically used psychotropic drugs with off-target DHCR7 inhibitory activity, including aripiprazole, cariprazine, trazodone, and haloperidol [22,43,163]. As it holds true for other previously discussed enzymes, there are also many other approved drugs that have been shown to affect the activity of DHCR7 in vitro, but the in vivo data is still lacking [164].

#### 4.2.1. SLOS Phenocopy Induced by Experimental Compounds

The two experimentally most well-established and commercially available DHCR7 inhibitors that have been shown to induce a SLOS-like phenocopy in adult animals are AY-9944 and BM 15.766. These cryptic names correspond to a cyclohexane-based non-competitive inhibitor and a piperazine-derivative competitive inhibitor of DHCR7, respectively [165,166]. While BM15.766 is DHCR7-specific, AY-9944 additionally exhibits a comparably less potent and competitive inhibition of sterol-14-reductase [167].

Although initially investigated as a cholesterol-lowering agent, AY-9944 was not pursued clinically because of its teratogenicity [168]. Nevertheless, its potent inhibition of DHCR7 has made it a widely used pharmacological tool for generating experimental models of SLOS. Due to its ability to permeate the BBB, it serves as an agent for inhibiting central nervous system cholesterol biosynthesis [13].

Evidence suggests that exposure to AY-9944 interferes with the sonic hedgehog (shh) signaling pathway in rodent embryos, thereby altering the normal ventral patterning of the cranial neural tube and contributing to developmental malformations [168]. Cholesterol is well-known to be of critical importance in the shh signaling pathway, since it covalently binds the shh ligand, preventing its leakage from the cell, and directly activates the G-protein-coupled downstream signaling molecules [169,170].

Beyond these early developmental effects, chronic postnatal exposure to AY-9944 (up to ten weeks) results in profound retinal degeneration in rats. This pathology correlates with lipid metabolism disturbances [168]. Specifically, one study identified five 7-DHC-derived oxysterols in the retinas of AY-9944-treated rats, including 3β,5α-dihydroxycholest-7-en-6-one, 4α- and 4β-hydroxy-7-DHC, 24-hydroxy-7-DHC, and 7-ketocholesterol [171].

Conversely, these pathological findings may contrast with observations of the compound’s utility in acute stress scenarios. Research indicates that when administered in the short term, AY-9944 can mitigate cellular damage rather than induce it. For instance, it has proven effective in inhibiting ferroptosis and lipid peroxidation in cancerous liver cells, and it acts as an agent for viral suppression. Through an interferon-mediated mechanism, AY-9944 promotes the clearance of viral infections, including vesicular stomatitis virus in cell culture and Zika virus in live models, suggesting that the timing and duration of exposure are critical determinants of its biological impact [22].

Similar to AY-9944, the synthetic piperazine derivative BM 15.766 was initially also investigated as a potential cholesterol-lowering drug [166,172]. As expected, BM 15.766 treatment also resulted in the accumulation of 7-DHC and 8-DHC in guinea pigs [173]. In the rat model, it caused severe fetal developmental abnormalities within the holoprosencephaly spectrum, such as brain anomalies, facial malformations, and pituitary agenesis. These malformations resulted directly from a reduction in maternal cholesterol levels accompanied by the accumulation of 7-DHC, thereby recapitulating the biochemical fingerprint of SLOS [174].

When administered to adult rats over a 14-day period, BM 15.766 effectively emulated the biochemical sterol profile characteristic of SLOS without typical teratogenic phenocopy. However, this was accompanied by significant hepatotoxicity, specifically manifesting as ballooning necrosis in pericentral hepatocytes [175].

In summary, the published data supports the claim that the discussed experimental compounds are able to reproduce the biochemical fingerprint of SLOS in adult animals. This fingerprint consists primarily of cholesterol depletion and accumulation of 7-DHC and 8-DHC, as well as secondary overproduction of oxidized 7-DHC derivatives, which are potent signaling molecules on their own.

#### 4.2.2. Clinically Used Drugs as Inadvertent DHCR7 Inhibitors

Recently, concerns have been raised about commonly used medications that show a clinically still underappreciated and understudied off-target inhibition of DHCR7 [23]. Many of these drugs belong to psychotropic medication and some of them are piperazine derivatives and share some chemical structural similarities [22]. To date, the most studied drugs with off-target DHCR7-inhibition are aripiprazole, cariprazine and haloperidol, belonging to the broader class of antipsychotic medication, and trazodone, an antidepressant and hypnotic agent.

While these drugs cause the accumulation of 7-DHC and by extension also of 7-DHC-derived oxysterols [176,177,178], in comparison to previously discussed experimental compounds AY-9944 and BM 15.766, they typically do not cause the full clinical manifestation of SLOS. In particular, the teratogenic effects are typically absent [162]. Observations have been made that the resulting phenotypes, rather than malformation, could be neurocognitive and behavioral instead, possibly resembling the deficits observed in autistic spectrum disorder [38,39,40,41].

Being approved psychotropic drugs, aripiprazole, cariprazine, haloperidol and trazodone readily cross the BBB. Therefore, they not only cause the elevation of 7-DHC in the peripheral tissues, but also in the brain, as demonstrated in vivo in murine models [179,180,181], zebrafish [182], as well as in human post-mortem brain samples in the case of trazodone [19]. It has been hypothesized that at least some of the beneficial psychotropic effects of these medications could be mediated by neurosteroid-like compounds generated from elevated 7-DHC [176].

Furthermore, maternal exposure to these drugs in rodents was associated with elevated 7-DHC and reduced desmosterol levels in offspring brains, mimicking the biochemical fingerprint of SLOS in intact, adult organisms, without developmental syndromes [166,174,175,183,184]. Prenatal exposure to haloperidol in rats additionally induced persistent hyperlipidemia in both first- and second-generation offspring. These findings suggest that prenatal haloperidol exposure may produce long-term metabolic alterations that can be transmitted across generations [185]. Moreover, chronic haloperidol treatment has been reported to impair sphingolipid synthesis in the CNS. These metabolic alterations suggest that the long-term adverse effects of haloperidol may arise from myelin damage in addition to dopamine receptor blockade [186].

In mouse embryos, exposure to cariprazine significantly increased 7-DHC levels in all embryonic organs, with the most pronounced effect observed in the brain. It also disrupted sterol metabolism across all animals examined, with the greatest alterations detected in the brains of pups heterozygous for *Dhcr7* born to heterozygous dams. Moreover, maternal cariprazine exposure increased toxic oxysterol levels in the brains of heterozygous offspring to concentrations approaching those observed in a mouse model of SLOS. Consistent with these preclinical findings, patients treated with the drug also exhibited elevated serum 7-DHC levels [21].

Another experimental study in lactating mice has shown that cariprazine is transferred to nursing offspring through maternal milk, where it accumulates in the brain and interferes with normal sterol metabolic pathways during early postnatal development [187].

Curiously, long-term treatment with aripiprazole has been associated with significant improvements in lipid profiles in patients with schizophrenia, whereas other antipsychotics typically precipitate metabolic syndrome and hypertriglyceridemia [188,189]. This is usually explained by the drug’s rather negligible antihistamine effects [190], and thus minimal increase in appetite, even though in a rodent model aripiprazole promoted hyperphagia and increased body weight, yet without elevated serum triglyceride concentrations and no induction of lipogenic gene expression [191]. Currently no study could have been retrieved that explicitly researched if the lipid metabolism under aripiprazole treatment could be influenced by the secondary accumulation of oxysterols, in particular their sulfated forms, which are known to downregulate the lipid biosynthesis [192].

To conclude, the available studies suggest that while clinically used psychotropic drugs with inadvertent DHCR7 inhibition might alone and/or synergistically replicate the biochemical fingerprint of SLOS (consisting of increased 7-DHC, 8-DHC and their derived oxysterols), they do not replicate the malformational aspects of the syndrome when given during embryonal development. In addition to 7-DHC and its derived oxysterols, whose pleiotropic biological functions has been briefly introduced in the previous paragraphs, the 8-DHC itself also possesses biological functions on its own, such as structural and functional suppression of shh signaling and downregulation of signaling cascades involved in cell survival [45,160,193]. Apart from that, accumulated 8-DHC has been observed to passively incorporate into the cell membranes instead of cholesterol molecules, altering the membrane biophysical properties, disrupting lipid rafts and consequently receptor-mediated transmembrane signaling [44,194]. Therefore, the secondary effects of these medications might in reality reflect a much more complex biochemical fingerprint than what the currently available data reveals.

### 4.3. DHCR24 Inhibition: The Desmosterol-Accumulation Fingerprint

DHCR24 is responsible for the final step of the Bloch cholesterol biosynthetic pathway, converting desmosterol to cholesterol [195]. Beyond this role, the enzyme facilitates cross-talk between pathways by converting various sterol intermediates from the Bloch pathway into their corresponding counterparts in the Kandutsch–Russell pathway [22]. Given these functions, the inhibition of DHCR24 disrupts cholesterol synthesis and leads to the accumulation of desmosterol (Table 1).

As an endogenous agonist of the liver X receptor (LXR), desmosterol regulates LXR signaling, a central pathway controlling lipid homeostasis and contributing to diverse disease mechanisms, including inflammatory disorders, atherosclerosis, cancer, diabetes mellitus, multiple sclerosis, nonalcoholic steatohepatitis, and viral infection progression [196]. Desmosterol exerts anti-inflammatory and lipid-regulatory effects in macrophages by activating LXR target genes and inhibiting SREBP signaling through SREBP cleavage-activating protein, a chaperone and sensor protein that controls cellular lipid metabolism. Since this activation is not observed in hepatocytes, increasing endogenous desmosterol levels may provide a tissue-specific, safer therapeutic approach for hypercholesterolemia and inflammatory conditions [197]. Therefore, pharmacological inhibition of DHCR24 emerges as a promising strategy for the selective, site-specific activation of LXR [196].

The currently best-established compounds with robust DHCR24 inhibitory effect attested in vivo are a cholesterol-lowering agent, MER-29, also known as triparanol, and somewhat clinically obsolete, first-generation antipsychotic drug, trifluoperazine [198,199]. Despite its initial clinical success, triparanol/MER-29 was ultimately withdrawn because of serious adverse effects, most notably the development of cataracts [200].

It has been found that blocking cholesterol production with triparanol disrupts normal cell cycle progression by inducing arrest at the G1 phase. Restoration of cholesterol through metabolic supplementation rescues this effect, demonstrating that the observed cell cycle inhibition is dependent on cholesterol depletion. This mechanism further corroborates the potential application of cholesterol-lowering compounds, such as triparanol, as therapeutic candidates for cancer treatment [201]. Building on these findings, subsequent studies have further demonstrated the anti-tumor potential of triparanol across multiple cancer models. In human lung, breast, liver, pancreatic, and prostate cancer cells, as well as melanoma cells, triparanol treatment inhibited cellular proliferation and promoted apoptosis. The ability of exogenous cholesterol supplementation to restore cell growth further supports the role of cholesterol depletion in mediating these effects. In addition to altering cholesterol-dependent processes, triparanol was also shown to suppress the shh pathway in cancer cells. These anti-cancer effects were further validated in vivo, where administration of triparanol reduced tumor growth in a mouse xenograft model of human lung cancer [202].

A study investigating the developmental effects of triparanol demonstrated that inhibition of DHCR24 activity resulted in a high incidence of limb malformations in rat fetuses following a single maternal oral exposure on gestational day 10. In addition to disrupting DHCR24-mediated cholesterol synthesis, triparanol was found to inhibit the conversion of Δ8- to Δ7-unsaturated sterols, resulting in the accumulation of desmosterol and zymosterol within embryonic tissues [203]. Further studies in rats have shown that early gestational exposure to triparanol results in significant teratogenic effects. The severity and range of developmental abnormalities were influenced by both the dose and route of administration, with the predominant defects characterized by a holoprosencephaly-like phenotype. Triparanol-induced inhibition of cholesterol synthesis leads to decreased cholesterol concentrations and increased desmosterol accumulation in maternal serum and embryonic tissues, contributing to these developmental abnormalities [204].

In contrast to triparanol, trifluoperazine’s inhibitory effect on DHCR24 was initially not immediately recognized, and, curiously, an early study in rabbits from the 1980s reported that long-term administration of this antipsychotic drug significantly increased serum cholesterol levels and was associated with cholesterol accumulation in the aorta [205]. Later, the drug was identified as the DHCR24 inhibitor, preventing the conversion of desmosterol into cholesterol [192,199]. Apart from the cholesterol-lowering effect, trifluoperazine reduced peripheral inflammation, hypothalamic microglial activation, and plasma TNF-α and IL-1β levels, while improving plasma glucose and insulin levels in obese mice fed a high-fat diet [206]. In a cuprizone-induced demyelination model in C57BL/6 mice, trifluoperazine attenuated demyelination and improved motor behavior. These protective effects are thought to be mediated through activation of the Nrf2 signaling pathway and inhibition of NF-κB signaling, thereby reducing oxidative stress [207].

Administration of trifluoperazine to pregnant rats and mice during organogenesis resulted in dose-dependent developmental toxicity. In rats, treatment induced cleft palate and micrognathia, whereas both species exhibited increased fetal resorptions. In mice, trifluoperazine also resulted in abortions; however, no overt teratogenic effects were observed [208]. Another study reported that male mice exposed to trifluoperazine exhibited increased micronucleus frequency, germ cell chromosomal aberrations, and sperm abnormalities [209].

In brief, the DHCR24 inhibition in vivo primarily leaves a biochemical fingerprint of cholesterol depletion and accumulation of desmosterol, a bioactive intermediate conferring potentially beneficial anti-inflammatory and lipid-regulatory effects (Table 1). During organogenesis, the inhibition causes variably pronounced malformation syndromes, whereas in adult organisms, cholesterol depletion might promote cell cycle arrest in proliferating tissues (including cancer), and, in the case of triparanol, to development of cataracts.

To conclude, pharmacological DHCR24 inhibition during pregnancy seems to fully recapitulate the phenotype of naturally occurring biallelic loss-of-function mutations in humans, resulting in a rare, severe autosomal recessive developmental disorder known as desmosterolosis [210]. The monoallelic loss of function was found to cause subtle transcriptional changes in the brain, altering early neuronal outgrowth and synaptogenesis [211]. It is, however, unknown if triparanol and/or trifluoperazine can induce these changes as well.

## 5. Limitations and Knowledge Gaps

Despite the increasing number of pharmacological tools available for perturbing cholesterol biosynthesis, important limitations currently restrict the interpretation of these models. First, pharmacological inhibition does not necessarily reproduce the biochemical or phenotypic consequences of a genetic defect. The clearest example is the proposed pharmacological model of MVK/MVD deficiency based on aminobisphosphonate-mediated inhibition of FPP-synthase. Because the intervention occurs downstream to MVK/MVD and may spare specific metabolic branches, its similarity to genetic MVK/MVD deficiency remains incomplete. Notably, no directly validated in vivo pharmacological model of MVK/MVD inhibition was identified in the literature reviewed here.

Second, compound-specific pharmacology represents a major source of uncertainty. Several inhibitors discussed in this review interact with targets outside the cholesterol biosynthetic network. This is particularly relevant for clinically used drugs whose sterol-related effects represent only one component of their pharmacology. Consequently, phenotypes observed after drug exposure should not immediately be assigned to cholesterol biosynthesis inhibition without appropriate pharmacological controls.

Third, the tissue specificity of pharmacological sterol perturbation remains incompletely characterized. This is particularly important for the CNS, where cholesterol metabolism is autonomous and substrate exchange across the BBB is restricted. Although several compounds clearly alter brain sterol metabolism, BBB penetration has not yet been established for all inhibitors discussed here. Similarly, the long-term consequences of sustained upstream feedback responses remain poorly characterized. For example, although CYP51A1 inhibition is expected to increase sterol-mediated suppression of HMGCR, the long-term consequences of this mechanism have not yet been directly examined in vivo.

Finally, several biochemical fingerprints remain incompletely defined. The consequences of systemic squalene accumulation following SQLE inhibition are inconsistent across experimental systems, while comprehensive measurements of oxysterols and non-sterol-isoprenoid are rarely performed alongside conventional cholesterol measurements.

These limitations and knowledge gaps emphasize that the current evidence base should be viewed as a framework for model development rather than a definitive catalog of validated pharmacological phenocopies.

## 6. Conclusions

Pharmacological inhibition of cholesterol biosynthesis represents a versatile experimental approach for studying the physiological functions of individual sterol and non-sterol intermediates.

As summarized in this review, currently available inhibitors target multiple enzymatic steps throughout the pathway, producing characteristic biochemical signatures that resemble distinct genetic sterolopathies, while allowing the reversible and dose-dependent modulation of sterol metabolism. Unlike constitutive genetic models, these pharmacological approaches permit investigation of cholesterol biosynthesis in adult organisms without the confounding effects of altered embryonic development. The principal value of pharmacological models may not lie in replacing genetic models, but in complementing them. Carefully characterized pharmacological fingerprints may reveal tissue-specific, temporal and exposure-dependent effects that are usually difficult to capture in constitutive genetic disorders.

Importantly, the reviewed studies show that the biological consequences of cholesterol biosynthesis inhibition extend well beyond cholesterol depletion itself. The site of inhibition, the alterations in non-sterol isoprenoid synthesis, accumulation of biologically active sterol intermediates, activation of compensatory feedback pathways and tissue-specific metabolic adaptations collectively shape the resulting phenotype. These observations emphasize that cholesterol biosynthesis should be viewed as an integrated metabolic signaling network, rather than a linear pathway. In that light, the drug-induced sterol disturbances should not simply be regarded as trivial side effects of medication, but rather as complex ramifications of the network perturbance.

## 7. Future Directions

Future research should move beyond measuring total cholesterol as the primary outcome of cholesterol biosynthesis inhibition and instead adopt comprehensive sterol profiling capable of quantifying pathway intermediates and non-sterol isoprenoid metabolites. Such approaches would enable standardized biochemical fingerprinting of pharmacological inhibitors and facilitate direct comparison between experimental models. Equally important should be systematic characterization of tissue-specific responses, particularly within the central nervous system, where autonomous cholesterol metabolism and restricted BBB transport may produce effects that differ substantially from those observed in peripheral organs.

Another important research priority should be the interaction between pharmacological inhibition and genetic susceptibility. Future studies should determine whether heterozygous carriers of pathogenic variants or individuals harboring common polymorphisms in genes coding for cholesterol biosynthesis enzymes exhibit increased vulnerability to drug-induced sterolopathies—and not only during fetal development, but also during childhood, adolescence and in geriatric age. Furthermore, the increasing recognition that numerous clinically approved medications interfere with cholesterol biosynthesis highlight the need for systematic pharmacovigilance. Apart from increasing safety, this could also help to identify novel opportunities for drug repurposing in neurological, inflammatory, metabolic and oncological diseases. In that light, integrating pharmacological models with multi-omics and precision medicine approaches may transform cholesterol biosynthesis inhibitors from experimental probes into powerful tools for personalized medicine.

Finally, we would like to suggest that our concept of biochemical fingerprints could be used translationally to establish standardized sterolomic diagnostic panels for each biosynthesis perturbation, incorporating the inhibited enzyme, primary accumulated intermediates, downstream sterols, oxysterols and relevant non-sterol isoprenoids. Such reference profiles would enable direct comparison between genetic and drug-induced sterolopathies and help to determine the extension of cholesterol biosynthesis signaling network perturbance in affected individuals.

## Figures and Tables

**Table 1:** Overview of the typical biochemical fingerprints of cholesterol biosynthesis enzyme defects, their characteristic secondary systemic effects, respective in vivo validated inhibitors, important off-target and confounding effects, as well as estimated model fidelity compared to corresponding sterolopathy or genetic model. ↗ Increase/Accumulation; ↘ Decrease/Depletion.

Target	Biochemical Fingerprint	Secondary Effects	Representative In Vivo Inhibitors	Important Off-Target/Confounding Effects	Model Fidelity
HMGCR	↘ mevalonate, ↘ downstream sterols, ↘ isoprenoids	↘ prenylation, ↘ ubiquinone, ↘ dolichol, ↗ HMG-CoA/protein HMGylation	Statins	Pharmacokinetic/tissue differences among statins	High fidelity compared to monoallelic *Hmgcr*^+/−^ knockouts and autoimmune anti-HMGCR myopathy (biallelic knockouts are not viable)
MVK/MVD *	↗ mevalonate, ↘ downstream sterols, ↘ FPP, ↘ GGPP	↘ prenylation, ↘ ubiquinone, ↘ dolichol, amplification of innate immunity/proinflammatory crisis	Aminobisphosphonates	Block is downstream of MVK/MVD, isopentenylation may be spared, poor BBB-penetration	Low to moderate fidelity compared to MVK/MVD-deficiency/hyperimmunoglobulinemia D (biallelic deficiency is not viable)
FDFT1	↗ FPP, ↘ squalene, ↘ downstream sterols	Altered lipid handling, ↗ dolichol, ↗ ubiquinone	Squalestatins, YM-53601	Hepatotoxicity/metabolic effects	Uncertain; moderate fidelity compared to in vitro monoallelic *Fdft1*^+/−^ silencing, in vivo data is lacking
SQLE	↗ squalene, ↘ downstream sterols	Relatively preserved non-sterol isoprenoids	NB-598, FR194738	Species- and compound-dependent effects	High fidelity compared to biallelic *Sqle*^+/−^ knockouts; moderate fidelity compared to monoallelic *Sqle*^+/−^ knockouts
LSS	↘ lanosterol, ↗ 2,3-oxidosqualene/shunt products	↗ epoxysterols, negative feedback on HMGCR	Ro 48-80701, MM0299	BBB-penetrance data incomplete	High fidelity compared to monoallelic *Lss*^+/−^ knockouts in vitro; no data on developmental effects
CYP51A1	↗ lanosterol, ↗ 14α-methyl-sterols	↗ epoxysterols, negative feedback on HMGCR	Azoles, Azalanstat	Multiple CYPs, azalanstat inhibits heme oxygenase	Uncertain, chronic exposure studies are lacking, monoallelic *Cyp51a1*^+/−^ knockouts reveal sex-specific metabolic disturbances
DHCR7	↗ 7-DHC, ↗ 8-DHC, ↘ cholesterol	↗ oxidized 7-DHC derivatives, SLOS-like biochemical signature	AY-9944, BM 15.766, Aripiprazole, Cariprazine, Trazodone, Haloperidol	Multiple, due to drug-specific receptor and other pharmacological effects	Experimental compounds: High fidelity compared to SLOS when given during in utero development, high biochemical fidelity when given to adult animals; Psychotropic drugs: high fidelity compared to monoallelic *Dhcr7*^+/−^ knockouts, high biochemic fidelity compared to SLOS, without dysmorphogenesis
DHCR24	↗ desmosterol, ↘ cholesterol	Altered Δ24-sterol balance, Anti-inflammatory and lipid-regulatory effects of desmosterol	Triparanol, Trifluoperazine	Calmodulin antagonism by trifluorperazine	High fidelity compared to desmosterolosis when given during in utero development, moderate fidelity compared to monoallelic *Dhcr24*^+/−^ knockouts when given to adult animals

* MVK/MVD-deficiency model via inhibition of farnesylpyrophosphate-synthase.

## Data Availability

No new data was created.

## Abbreviations

The following abbreviations are used in this manuscript:

BBB	Blood–Brain Barrier
CNS	Central Nervous System
CYP51A1	Lanosterol-14α-demethylase
DHCR7	7-dehydrocholesterol-reductase
DHCR24	24-dehydrocholesterol-reductase
FPP	Farnesyl Pyrophosphate
FDFT1	Squalene synthase
GGPP	Geranylgeranyl Pyrophosphate
GTP	Guanosine Triphosphate
HMG-CoA	3-hydroxy-3-methylglutaryl-co-enzyme-A
HMGCR	HMG-CoA-reductase
LSS	Lanosterol-synthase
LXR	Liver X receptor
MVK	Mevalonate kinase
MVD	Mevalonate decarboxylase
SLOS	Smith–Lemli–Opitz Syndrome
SQLE	Squalene epoxidase
SREBP	Sterol Regulatory Element-Binding Proteins
7-DHC	7-dehydrocholesterol
7-DHD	7-dehydrodesmosterol
8-DHC	8-dehydrocholesterol
